# Gain-of-Function Mutations in the K_ATP_ Channel (KCNJ11) Impair Coordinated Hand-Eye Tracking

**DOI:** 10.1371/journal.pone.0062646

**Published:** 2013-04-23

**Authors:** James S. McTaggart, Ned Jenkinson, John-Stuart Brittain, Siri A. W. Greeley, Andrew T. Hattersley, Frances M. Ashcroft

**Affiliations:** 1 Department of Physiology, Anatomy and Genetics, University of Oxford, Oxford, Oxfordshire, United Kingdom; 2 Nuffield Department of Surgical Sciences, University of Oxford, Oxford, Oxfordshire, United Kingdom; 3 Centre of Excellence in Personalized Healthcare, Institute of Biomedical Engineering, Department of Engineering Science, University of Oxford, Oxford, Oxfordshire, United Kingdom; 4 Department of Neurological Surgery, John Radcliffe Hospital, University of Oxford, Oxford, Oxfordshire, United Kingdom; 5 Kovler Diabetes Center, University of Chicago, Chicago, Illinois, United States of America; 6 Institute of Biomedical and Clinical Science, Peninsula Medical School, University of Exeter, Exeter, Devon, United Kingdom; Sackler Medical School, Tel Aviv University, Israel

## Abstract

**Background:**

Gain-of-function mutations in the ATP-sensitive potassium channel can cause permanent neonatal diabetes mellitus (PNDM) or neonatal diabetes accompanied by a constellation of neurological symptoms (iDEND syndrome). Studies of a mouse model of iDEND syndrome revealed that cerebellar Purkinje cell electrical activity was impaired and that the mice exhibited poor motor coordination. In this study, we probed the hand-eye coordination of PNDM and iDEND patients using visual tracking tasks to see if poor motor coordination is also a feature of the human disease.

**Methods:**

Control participants (n = 14), patients with iDEND syndrome (n = 6 or 7), and patients with PNDM (n = 7) completed three computer-based tasks in which a moving target was tracked with a joystick-controlled cursor. Patients with PNDM and iDEND were being treated with sulphonylurea drugs at the time of testing.

**Results:**

No differences were seen between PNDM patients and controls. Patients with iDEND syndrome were significantly less accurate than controls in two of the three tasks. The greatest differences were seen when iDEND patients tracked blanked targets, i.e. when predictive tracking was required. In this task, iDEND patients incurred more discrepancy errors (p = 0.009) and more velocity errors (p  = 0.009) than controls.

**Conclusions:**

These results identify impaired hand-eye coordination as a new clinical feature of iDEND. The aetiology of this feature is likely to involve cerebellar dysfunction. The data further suggest that sulphonylurea doses that control the diabetes of these patients may be insufficient to fully correct their neurological symptoms.

## Introduction

Adenosine triphosphate (ATP)-sensitive potassium (K_ATP_) channels are important metabolic sensors that link cell metabolism to electrical activity in neurones, pancreatic beta-cells and many other cell types [1]. They do so by sensing changes in intracellular nucleotides such as ATP, which closes the channel and reduces the K_ATP_ current. Gain-of-function mutations in either the Kir6.2 (*KCNJ11*) or SUR1 (*ABCC8*) subunits of the channel that impair the inhibitory effects of ATP cause a rare genetic form of diabetes that presents shortly after birth (permanent neonatal diabetes mellitus or PNDM). About 30% of patients also experience muscle hypotonia, delayed speech and motor milestones, and hyperactivity [2], a condition known as iDEND syndrome (iDEND). The neurological symptoms have not yet been fully characterised, and other problems may also be present.

The diabetes of both PNDM and iDEND patients is well controlled by oral sulphonylurea drugs, which close K_ATP_ channels. By contrast, improvements in the neurological symptoms are more variable: some patients show improvements in motor function and cognitive development [3], [4], whereas others do not [3].

We generated a transgenic mouse in which K_ATP_ channels harbouring an iDEND-causing mutation were expressed only in neurones [5]. These mice showed impaired motor coordination but, as expected, lacked a diabetic phenotype. The electrical activity of their cerebellar Purkinje neurones was markedly less than control mice, raising the possibility that cerebellar function is also impaired in iDEND patients. To test this hypothesis, we compared the hand-eye coordination of iDEND patients, PNDM patients and matched controls using a task in which moving visual targets were tracked with a joystick-controlled cursor. Such coordinated hand-eye tracking is dependent on cerebellar processing [6].

## Materials and Methods

### Ethics Statement

Studies were conducted in accordance with the Declaration of Helsinki. The research was approved by the Institutional Review Board (IRB) of the University of Chicago, USA. Informed written consent was received from participants (or their parents/guardians, in the case of minors) before carrying out any experiments.

### Participants

Patients with activating mutations in the *KCNJ11* gene were recruited along with non-affected members of their family (who acted as controls). All patients with PNDM and iDEND were being treated with sulphonylurea drugs at the time of testing, although we lack details of the agent and dosage for each individual. The age at which the patients had been transferred to oral therapy, and the duration of their treatment also differed. Some patients had been hospitalised for hypoglycaemic attacks at some point in their life.

For data analysis, PNDM and iDEND patients were matched with a non-diabetic individual of a similar age and, where possible, the same sex (Table S1, Table S2). (Note the iDEND patient from pair 4 did not complete task 3.) All individuals included in statistical analyses showed good attentional control when carrying out the task: they did not look away from the screen, they did not talk and they sat still. Three patients with iDEND were unable to carry out the tasks as they showed high levels of attention deficit. All were male, had V59 M mutations, and were aged 5, 6, and 9 years old. Other children, of a similar age, who did not have K_ATP_ channel mutations were able to complete the task.

### Tasks

Participants used a custom-built joystick to control a cursor presented on the 17″ screen of a laptop [7]. The joystick had a built-in arm-rest and target tracking only required movement of the wrist. All participants used their right hand to control the joystick. The target and cursor moved in a straight line, horizontally across the middle of the screen. Participants were requested to track the target as it moved from left to right and back again. Movement of the target was programmed, and movement of the cursor recorded, by a custom-made application built in Labview (National Instruments). Data were acquired via a USB data acquisition device (National Instruments USB-6008) and sampled at 50 Hz. Data were analysed using custom-built routines in Matlab (Mathworks).

Three tasks were undertaken, each composed of 12 tracks in total - 6 rightward and 6 leftward. Each track was 4 s long and the target paused for 1.4 s at either end of the tracking run before the next track commenced. The tasks were undertaken in sequential order. In the first task (task 1), the target moved at a constant speed from left to right and right to left. In task 2 the target moved in a sinusoidal manner - accelerating and decelerating symmetrically during each track. In task 3 the target moved at constant speed but the visual presentation of the target was switched off (it was blanked) during the middle third of each track. Participants were asked to continue 'as if the target was still there'.

### Analysis

The first sweep of each task was omitted from analysis because the target was not visible when the task began, so participants were often in the wrong starting position. The final sweep was also removed so that the number of leftward rightward sweeps were equal. Thus, ten sweeps (5 leftward, 5 rightward) were analysed for each participant. Both controls and patients tended to perform better on rightward sweeps (data not shown). The reason for this difference is unclear, but may be due to differences in the hand-eye coordination for flexion versus extension of the wrist. The stationary periods were also removed so that only tracking performance was assessed.

The following parameters were analysed:

- Discrepancy error: The difference between the position of the target and the cursor was calculated for each sampled point. The standard deviation of these differences (calculated per participant) was called the discrepancy error. Discrepancy errors indicated how accurately the participants tracked the target.
- Velocity error: The difference between successive points in the target track, and successive points in the cursor track was calculated. These differences were differentiated and the standard deviation of these differentiated errors was called the velocity error. Velocity errors indicated how well the participants matched its speed.

The Kruskall-Wallis ANOVA based on ranks was used to assess the significance of the results. Each analysis looked for differences between the four groups: PNDM patients, iDEND patients, and the two groups of matched controls. Where significant differences were found, pairwise comparisons were carried out, using Mann-Whitney U-tests, to compare PNDM and iDEND patients with their respective controls in order to identify which groups were different.

## Results

**Figure 1:**
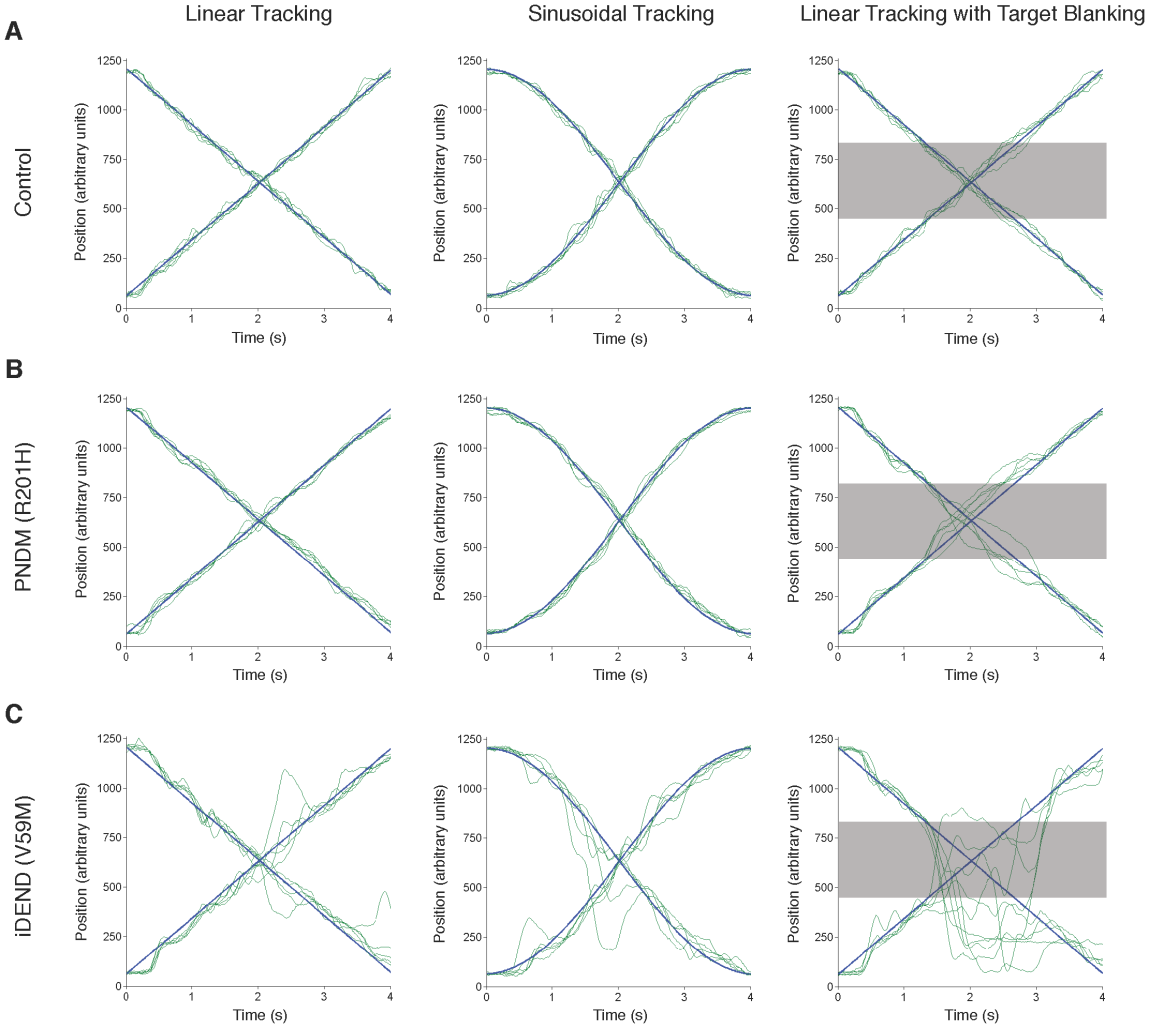
Representative traces from tracking tasks completed by a control participant, and patients with PNDM and iDEND. Panels show the superimposition of 5 rightward (positive slope) and 5 leftward (negative slope) tracks from a control subject (A), a PNDM patient (R201 H mutation) (B), and an iDEND patient (V59 M mutation) (C). The person who provided the traces shown in (A) was the matched control of the iDEND patient whose traces are shown in (C). Participants tracked targets that moved at constant velocity (first column), variable velocity – accelerating and decelerating smoothly (second column), and constant velocity but with the visual presentation of the target blanked during the period indicated by the grey bar (third column). Blue lines indicate the movement of the target. Green lines indicate the movement of the cursor, which was controlled by the participant.

Representative traces from a control individual, a PNDM patient and an iDEND patient for each of the three tasks are shown in Figure 1.

**Figure 2:**
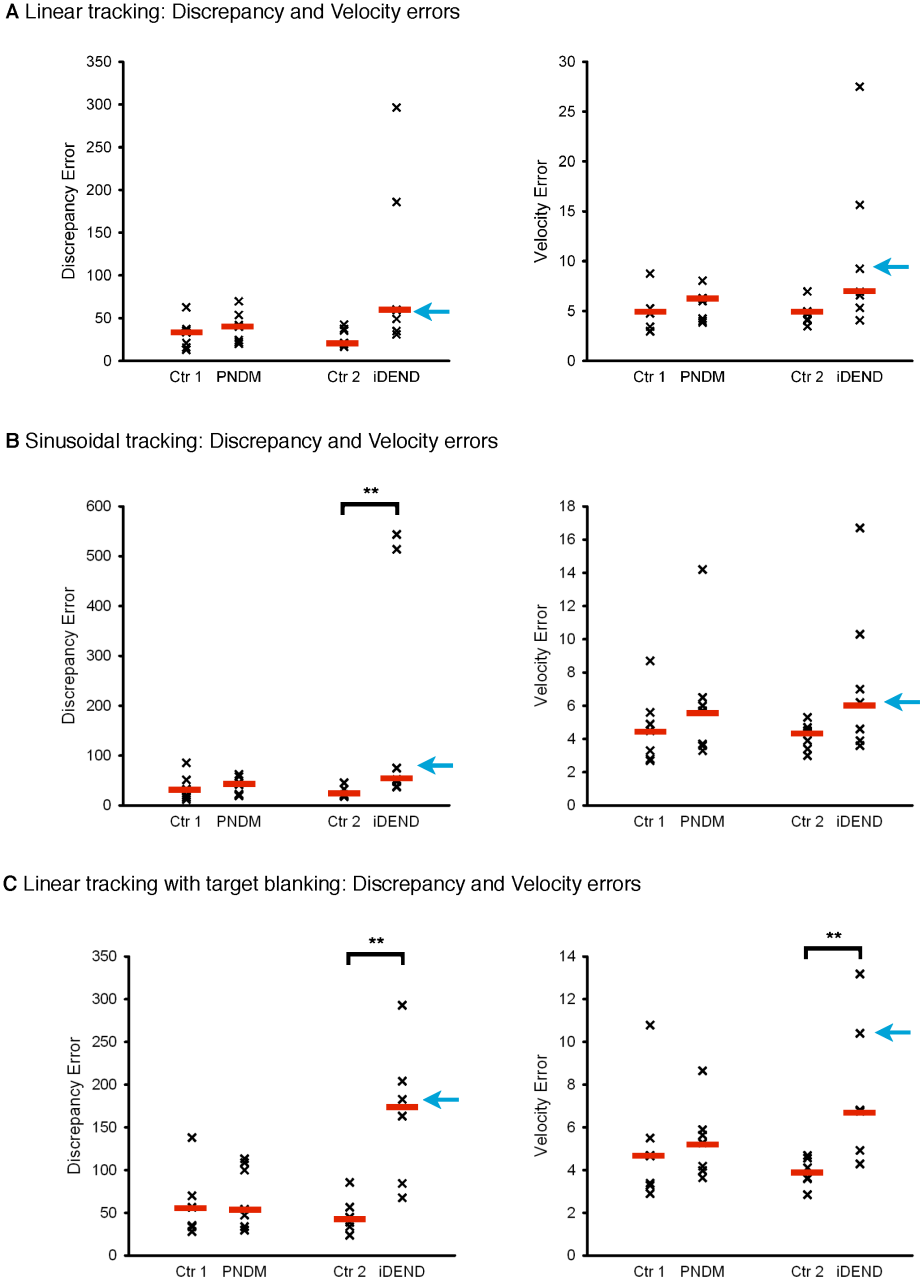
Tracking performance of patients with PNDM and iDEND on three different tasks. Scatter plots showing the performance of participants who tracked: a target moving at constant velocity (linear tracking) (A); a target moving with variable velocity (sinusoidal tracking) (B); and a target moving at constant velocity which was blanked during the middle third of each sweep (C). In each case the left panel shows discrepancy errors and the right panel velocity errors. (A) Data for PNDM (n = 7) and iDEND (n = 7) patients and their matched control, as indicated. There were no significant differences in the two error types between the groups (Kruskal-Wallis tests; p = 0.06 for discrepancy errors, p = 0.07 for velocity errors). (B) Data for PNDM (n = 7) and iDEND (n = 7) patients and their matched controls, as indicated. Patients with iDEND incurred significantly higher discrepancy errors than controls, but PNDM patients were not affected. **, p<0.01, post-hoc Mann-Whitney U-test. There were no significant differences in the velocity errors between the groups (Kruskal-Wallis test). (C) Data for PNDM (n = 7) and iDEND patients (n = 6) and their matched controls, as indicated. The target was blanked during the middle third of each sweep. Patients with iDEND were significantly less accurate than controls but PNDM patients were not different. **, p<0.01, post-hoc Mann-Whitney U-tests. In all figures the red bars indicate the median error and the blue arrows indicate data points for the iDEND patient shown in Figure 1.

When the target moved at constant velocity, there was no significant difference in the median discrepancy or velocity errors between iDEND patients and controls, although the trends neared significance (Figure 2A). On the sinusoidal tracking task, iDEND patients tracked significantly less accurately than controls (p = 0.002), but there was no significant difference in their velocity errors (Figure 2B). The greatest impairments were seen in the linear tracking with target blanking task (Figure 2C): both discrepancy and velocity errors of iDEND patients were higher than controls on this task (p = 0.009 in both cases). There was no difference between PNDM patients and controls on any of the three tasks (Figure 2).

**Figure 3:**
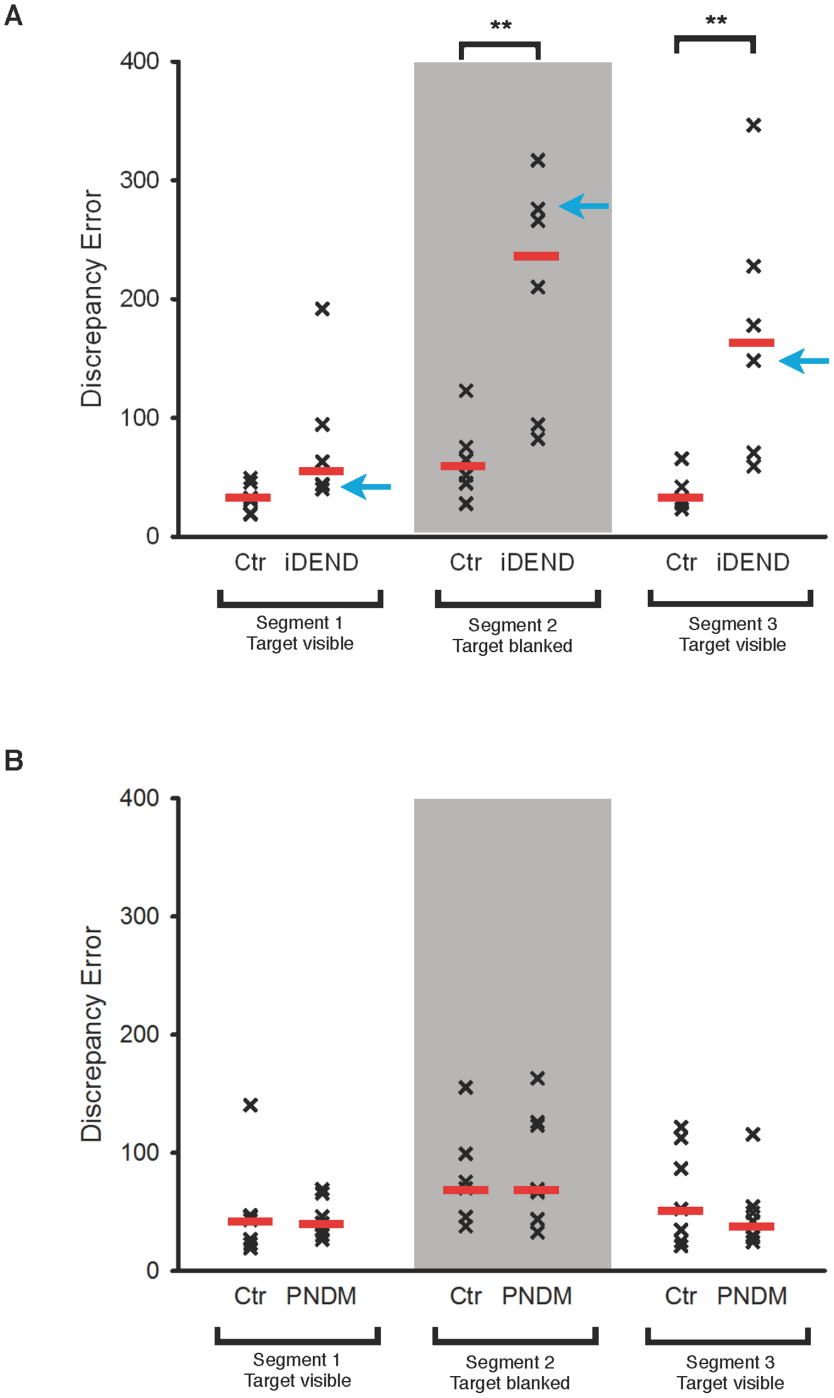
Comparison of visually-guided and blanked tracking performance of patients with PNDM and iDEND. Scatter plots showing discrepancy errors of iDEND patients (A, n = 6) and PNDM (B, n = 7) patients and their matched controls (Ctr), as indicated, on the linear tracking task with target blanking. The visually guided (first and last) and blanked (middle) segments of this task were analysed separately. Red bars indicate the median error. Discrepancy errors of iDEND patients were significantly higher than controls in segments 2 and 3. **, p<0.01 post-hoc Mann-Whitney U-tests. There were no differences between PNDM patients and controls in any of the three segments (Kruskal-Wallis test).

The linear tracking with target blanking task comprised three ‘segments’. The first and last segment probed visually guided tracking, and the middle segment assessed blanked tracking. When these segments were analysed separately there was no significant difference in discrepancy errors between iDEND patients and their controls for segment 1 (Figure 3A). This is consistent with the results of the linear tracking task (Figure 2A). By contrast, the discrepancy errors of iDEND patients were significantly higher than their controls in the blanked segment (p = 0.009). In segment 3, iDEND patients also performed significantly worse (p = 0.004), probably because they were already off-target after segment 2. In contrast to iDEND patients, PNDM patients performed as well as controls on all three segments (Figure 3B).

## Discussion

Our results show that patients with iDEND syndrome have impairments in coordinated hand-eye tracking. The fact that the deficits were most obvious when tracking blanked targets is consistent with the increased difficulty of this task, which requires greater activation of the cerebellum and cerebrum than tracking visible targets [8]. The data reveal that predictive movement is particularly affected in iDEND individuals.

By contrast, there were no significant differences in discrepancy or velocity errors between patients with PNDM and their matched controls on any of the three tracking tasks. There was no difference in glycaemic control (or in the frequency of hypoglycemic attacks) between iDEND and PNDM patients, either on insulin or following transfer to sulphonylurea therapy. Thus the difference in hand-eye coordination must be a novel feature of iDEND and not a secondary consequence of neonatal diabetes.

Although the tasks employed were not specific to the cerebellum, the performance of iDEND patients was particularly poor when tracking blanked targets. Cerebellar processing is important for this type of coordination [9], suggesting that cerebellar dysfunction may be present in iDEND and that therapies directed at improving cerebellar function may be helpful. It is possible that inaccurate hand-eye coordination may also contribute to the developmental delay of iDEND patients: for example, by making writing more difficult. It might also contribute to their poor motor control [10] and the fact that after they have finally learnt to walk, they fall over more frequently.

The tracking performance of iDEND patients was impaired even though they were on sulphonylurea therapy, which indicates that existing sulphonylurea therapy is not fully effective at treating the neurological symptoms. This may be due to irreversible developmental changes caused by the mutation (prior to drug therapy), or may suggest that the drug fails to achieve concentrations high enough to shut hyperactive K_ATP_ channels in brain circuits required for hand-eye coordination. Cerebral concentrations of the drug are difficult to ascertain but are likely to be lower than plasma levels due to poor penetrance across the blood-brain barrier, and efflux mechanisms that pump sulphonylureas out of the CSF [11].

Unlike iDEND patients, PNDM patients were not significantly impaired compared to matched controls. This may be a consequence of the lesser reduction in ATP sensitivity produced by PNDM mutations. Because all patients had diabetes, it seems that a much larger increase in K_ATP_ current is required to produce effects on neuronal activity than on pancreatic beta-cell function, probably due to the different complement of ion channels these cell types possess: unlike neurones, the resting membrane potential of the beta-cell is largely dependent on K_ATP_ channel activity.

In summary, we have identified a novel clinical feature of iDEND that may contribute to the delayed development of iDEND patients. These results, and those from the mouse model [5], suggest that cerebellar dysfunction may be a feature of iDEND, although further experiments are needed to support this interpretation. Because PNDM patients exhibited no obvious cerebellar deficits, it appears that a small reduction in K_ATP_ channel ATP sensitivity, and presumably thus a small increase in K_ATP_ current, is not sufficient to affect the electrical activity of neurones involved in predictive movements, whereas a much larger increase in K_ATP_ current can adversely affect neuronal function. Our data also illustrate the value of mouse models, which when combined with human studies can illuminate and identify novel features of the disease not previously reported in humans.

## Supporting Information

Table S1 Details of PNDM patients and their matched controls.
(PDF)

Table S2 Details of iDEND patients and their matched controls.
(PDF)
