# Supplementary material for: Gain-of-Function Mutations in the KATP Channel (KCNJ11) Impair Coordinated Hand-Eye Tracking
Source: PLoS One. 2013 Apr 23;8(4):e62646. doi: 10.1371/journal.pone.0062646 (PMC3633835; doi:10.1371/journal.pone.0062646)
Supplement: Table S1 — Details of PNDM patients and their matched controls. (PDF) [file pone.0062646.s001.pdf]

TABLE S1

Pair	Group	Mutation	Age (years)	Sex
1	Control	wt	9	F
	PNDM	R201H	9	F
2	Control	wt	10	M
	PNDM	R201H	9	M
3	Control	wt	7	F
	PNDM	R201H	7	F
4	Control	wt	28	M
	PNDM	R201H	29	M
5	Control	wt	24	M
	PNDM	R201H	18	M
6	Control	wt	25	F
	PNDM	R201H	20	F
7	Control	wt	6	M
	PNDM	E322K	7	M
Pooled	Control		Mean = 16	4M, 3F
	PNDM		Mean = 14	4M, 3F
