# Supplementary material for: Gain-of-Function Mutations in the KATP Channel (KCNJ11) Impair Coordinated Hand-Eye Tracking
Source: PLoS One. 2013 Apr 23;8(4):e62646. doi: 10.1371/journal.pone.0062646 (PMC3633835; doi:10.1371/journal.pone.0062646)
Supplement: Table S2 — Details of iDEND patients and their matched controls. (PDF) [file pone.0062646.s002.pdf]

TABLE S2

Pair	Group	Mutation	Age (years)	Sex
1	Control	wt	25	F
	iDEND	V59M	13	F
2	Control	wt	27	F
	iDEND	V59M	20	F
3	Control	wt	15	M
	iDEND	V59M	17	F
4 *	Control	wt	8	F
	iDEND	V59M	9	M
5	Control	wt	10	M
	iDEND	R201C	9	F
6	Control	wt	30	F
	iDEND	R201C	33	F
7	Control	wt	35	F
	iDEND	R201S	36	M
Pooled	Control		Mean = 21	2M, 5F
	iDEND		Mean = 20	2M, 5F

* Patient with iDEND only completed tasks 1 & 2.
